# Effectiveness of Game-Based Training of Selective Voluntary Motor Control in Children With Upper Motor Neuron Lesions: Randomized Multiple Baseline Design Study

**DOI:** 10.2196/47754

**Published:** 2024-11-18

**Authors:** Annina Fahr, Andrina Kläy, Larissa S Coka, Hubertus J A van Hedel

**Affiliations:** 1 Swiss Children's Rehab University Children's Hospital Zurich Affoltern am Albis Switzerland; 2 Children's Research Center University Children's Hospital Zurich University of Zurich Zurich Switzerland; 3 Institute for Biomechanics ETH Zurich Zurich Switzerland

**Keywords:** neurorehabilitation, single-case design, interactive computer play, cerebral palsy, surface electromyography, motor control, mirror movements, involuntary movements

## Abstract

**Background:**

Selective voluntary motor control (SVMC) is the ability to control joint movements independently. Impairments in SVMC can affect functional activities, but only a few interventions directly target SVMC. Therefore, we developed a game-based intervention for children with upper motor neuron lesions to improve SVMC. The intervention trained selective activation of a muscle or joint movement while providing immediate feedback about involuntarily occurring muscle activations or movements in another joint. The intervention was provided in a playful manner with a custom-made game environment and a technology-based interface to capture muscle activation or joint movements.

**Objective:**

This study aimed to investigate the effectiveness of this game-based intervention and explore treatment response–related factors in children with impaired SVMC undergoing inpatient neurorehabilitation.

**Methods:**

We conducted a single-case research study with a randomized, nonconcurrent, multiple baseline design. The study consisted of a random-length baseline phase where no SVMC-specific intervention was provided and an intervention phase with additional SVMC training. Concurrently in both phases, children attended their individual multimodal rehabilitation program at our clinic, Swiss Children’s Rehab. During the intervention phase, participants completed ten 45-minute sessions with our game-based SVMC training. SVMC was measured repeatedly throughout both phases and at the 3-month follow-up with a short custom-made assessment.

**Results:**

Eighteen children with reduced SVMC from upper motor neuron lesions participated in the study. The mean age of the children was 12.7 (SD 2.9) years, and they mostly had spastic cerebral palsy. A linear mixed-effects model revealed a significant trend (*P*<.001) for improved SVMC already in the baseline phase. This trend did not change significantly (*P*=.15) when the game-based SVMC training was introduced in the intervention phase, suggesting no additional improvements due to the SVMC training. Although we could not find an overall treatment effect, we could explain 89.4% of the total random variation of the treatment effect by patient and therapy characteristics. Children with spasticity in the trained movement (20.1%), and those who trained the more affected side (23.5%) benefited most from the intervention. At the 3-month follow-up, SVMC had deteriorated compared to the end of the intervention but was still better than at the beginning of the study.

**Conclusions:**

The regular concomitant rehabilitation program already yielded improvements in SVMC, while the game-based SVMC training showed no additional effects. Although the intervention did not show a group effect, we could identify patient and therapy characteristics that determine who is likely to profit from the intervention.

**Trial Registration:**

German Clinical Trials Register DRKS00025184; https://tinyurl.com/msnkek9b

## Introduction

### Background

Children and adolescents with upper motor neuron lesions because of a congenital or acquired brain injury, for example, spastic cerebral palsy (CP) or stroke, exhibit a variety of motor impairments. Impaired selective voluntary motor control (SVMC) is one common motor sign and refers to the loss of independent control of joint movements [1]. Reduced SVMC is defined as “the impaired ability to isolate the activation of muscles in a selected pattern in response to demands of a voluntary posture or movement” [2]. Consequences of reduced SVMC are impaired motor control and the occurrence of nonselective, involuntary movement patterns accompanying intended movements. These involuntary movements include mirror movements, that is, simultaneous movements in the contralateral joint, extra movements within the same limb, for example, synergistic flexion patterns of several joints, or involuntary movements in other joints [1,3,4].

Loss of SVMC contributes substantially to the disability of the patients and is a core impairment for children with CP as it can negatively influence other body functions and activities [5]. For example, children showing mirror or extra movements have more impaired manual abilities. They experience more difficulties with upper extremity tasks in daily life and need more time for bimanual tasks [3,6]. In addition, it has been repeatedly shown that impairment in SVMC influences gross motor function substantially more than other common impairments in children with CP [7-11]. Furthermore, impaired SVMC negatively impacts the walking ability (eg, gait velocity) of children with spastic CP [12].

Treatment strategies that directly target impaired selective control are rare, although the relevance of SVMC has been recognized [13]. For example, some studies applied robot-assisted training and computer games to improve ankle joint control [14-16]. In other studies, participants tried to reduce wrist flexor-extensor cocontraction or train selective ankle dorsiflexion by controlling a game with surface electromyography (sEMG) signals [17,18]. However, these studies were limited as they targeted only one specific joint or a single aspect of SVMC (eg, cocontraction).

To evaluate SVMC-specific interventions, adequate outcome measures are required. Most clinical SVMC assessments are easy to conduct observer-based tests [19-22]. However, their ordinal scales include only a few levels, which might negatively influence the responsiveness. Therefore, more complex kinematic or neurophysiological methods were suggested to improve the quantification of SVMC [23,24]. Following these recommendations, we have developed 2 approaches. First, we record sEMG while the patient performs clinical tests, and the second is a playful assessment that uses inertial measurement units [25-27].

The current opinion is that muscle synergies can provide insight into the neuromuscular control of children with CP [28]. A set of muscles commonly activated together is identified from sEMG signals during functional tasks (eg, walking) with computational techniques. The number of synergies reflects how refined the motor control is. A low number of synergies indicates greater muscle coactivation and thus, lower SVMC. Leg muscle activity of children with CP during walking revealed fewer synergies compared to healthy peers. The reduced synergy complexity was also related to their walking abilities and clinical SVMC measures [28]. However, altering neuromuscular control mechanisms with interventions remains a challenge. Neither short-term biofeedback nor common treatments for children with CP could evoke changes in muscle synergies, despite observing kinematic changes in the gait pattern [29,30]. Hence, treatment strategies directly targeting impaired control likely have the highest potential to improve SVMC [11,30].

### Objective

We have developed a game-based intervention for children with upper motor neuron lesions to specifically improve SVMC [31]. The principle of the intervention is that it trains accurate joint movement control while simultaneously providing immediate feedback about the occurrence of involuntary movements (via an alarm sound). The intervention appeared feasible and motivating for children to practice. Therefore, the primary aim of this clinical trial was to investigate the effectiveness of this game-based intervention in improving SVMC in children and youths with upper motor neuron lesions in a randomized multiple baseline design study. The secondary aims were to explore whether the treatment response was related to patient characteristics, investigate the effect of the intervention on secondary outcomes (clinical SVMC measures, muscle strength, and functional independence), and measure whether any changes were maintained 3 months after the intervention. The detailed study protocol also included further aims not covered here [32].

## Methods

### Participants

The inclusion criteria were as follows: (1) acquired or congenital brain injury that caused an upper motor neuron lesion, (2) aged between 6 and 20 years, (3) impaired SVMC of the target joint, indicated by scores 0 or 1 in the validated German version of the Selective Control Assessment of the Lower Extremity (SCALE) [21] or scores 1 or 2 in the German Selective Control of the Upper Extremity Scale (SCUES) [33], (4) Manual Muscle Test (MMT) [34] score≥2 of the target joint, (5) pain-free movement of the involved joints, and (6) ability to understand and follow 2-step commands, for example, “close your eyes and clap your hands,” to guarantee the ability to handle 2 instructions during the intervention, that is, move one joint without moving another.

The exclusion criteria comprised the following: (1) ataxia or primary dyskinetic movement component (dystonia, athetosis, and chorea) in the involved joints, (2) surgery or treatment with Botox during the last 3 months in one of the involved joints, (3) uncorrected visual or auditory limitations that hindered playing the game, (4) skin lesions that prevented the correct placement of sensors or electrodes, (5) inability to play the game for any other reason, or (6) noncompliance with the instructions.

We characterized the participants by age, diagnosis, and the more affected side. Furthermore, therapists not involved in the project assessed the MMT and the Modified Ashworth Scale (MAS) [35] before starting the study. Certified nurses assessed the cognition domain of the functional independence measure for children (WeeFIM) [36]. Specifically for children with CP, we included the Manual Ability Classification System (MACS) [37] and the Gross Motor Function Classification System (GMFCS) [38] to quantify upper and lower limb disability, respectively.

We recruited patients from our clinic, Swiss Children’s Rehab, from June 2021 to May 2022. We provided physical and occupational therapists with the main inclusion and exclusion criteria and asked them to identify children with reduced SVMC who exhibited involuntary movements during therapy. Therapists informed all potential participants and parents about the study and requested permission for a member of the research team to contact them. A researcher then reached out to the child and parents, providing verbal information about the study, confirming the child’s eligibility, and furnishing written information. After obtaining consent, the researcher carefully reviewed the inclusion criteria for the child.

### Ethical Considerations

We informed participants and their legal guardians verbally and in writing (children younger than 10 years only verbally). All participants had to provide verbal informed consent before enrollment. All legal guardians and youths aged 14 years and older provided written informed consent. Children and adolescents who participated in the study received a small gift (value CHF 15 [approximately US $16]) at the end of the intervention and at follow-up. Our study met the necessary guidelines, and the cantonal ethics committee of Zurich approved it (approval number PB 2021–00791). Before patient recruitment started, we listed the trial in the German Clinical Trials Register (DRKS00025184), registered on April 28, 2021.

### Study Design

We conducted a single-case study and applied a randomized, nonconcurrent, multiple baseline design across participants consisting of a baseline and intervention phase [39]. Single-case experimental designs are characterized by repeated outcome assessments throughout the study phases to describe and understand the variability between participants and by participants acting as their control [40]. Therefore, such research designs could be particularly suitable for small and heterogeneous samples like in pediatric rehabilitation [41]. In the particular case of a multiple baseline design across participants, the start of the intervention phase for the participants is staggered across time, that is, the length of the baseline phase differs (randomly) between participants. Nonconcurrent implies that not all participants are enrolled in the study at the same time. The goal of this design is to demonstrate that change in the outcome occurs when and only when the intervention is introduced to the participant. A random baseline length strengthens the internal validity because it ensures that the treatment response is not a result of the timing of events but only occurs when the intervention is introduced [39,40].

**Figure 1:**
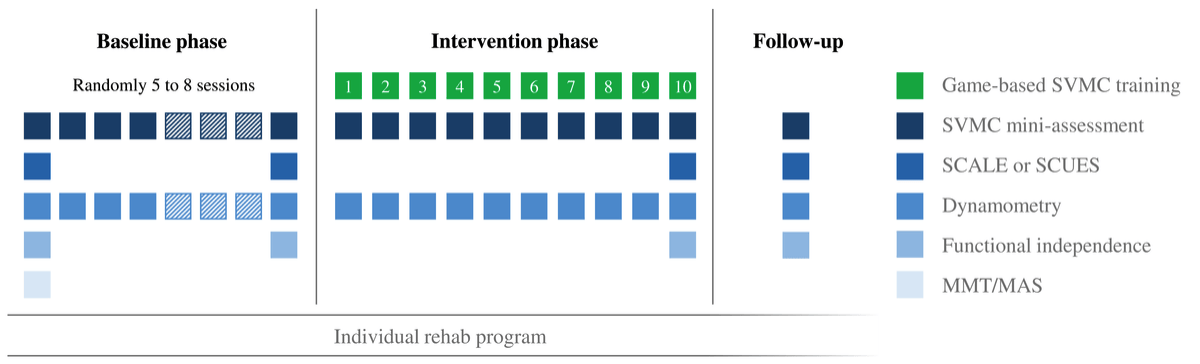
The flowchart outlines the daily SVMC assessment and training sessions during the study with a multiple baseline design and which assessments were completed at each visit. Participants (children with reduced SVMC) attended a multimodal rehabilitation program concomitant to the baseline and intervention phases. MAS: Modified Ashworth Scale; MMT: Manual Muscle Test; SCALE: Selective Control Assessment of the Lower Extremity; SCUES: Selective Control of the Upper Extremity Scale; SVMC: selective voluntary motor control.

The baseline phase (Figure 1), where no SVMC intervention was provided, randomly comprised 5 to 8 short assessment sessions of the primary outcome (custom-made SVMC measurement described below). Afterward, participants completed ten 45-minute intervention sessions with our game-based SVMC training. Each session ended with the same short assessment as during the baseline. Both study phases ran concurrently to intensive multimodal rehabilitation at Swiss Children’s Rehab. The rehabilitation program included physical, occupational, or speech and language therapy, as well as robotics and sports therapy (including endurance, strength training, and sports groups). These therapies were arranged according to the patient’s individual needs. The sessions were scheduled every weekday unless there were organizational limitations (eg, coordination with other treatments, school lessons, facilities, and staff).

The schematic study design is shown in Figure 1 and includes additional assessments that were mostly completed at the beginning and end of each phase. A follow-up appointment took place 12 weeks after completing the intervention. The SVMC intervention usually ended shortly before the participants were discharged from the rehabilitation program. After discharge, they could attend regular outpatient therapies during the follow-up period (usually 1-2 sessions of physiotherapy or occupational therapy per week). After inclusion, the baseline length was randomized for each participant between 5 and 8 sessions with an urn scheme for balanced distribution using a custom-made script in R (version 4.1.2, R Foundation for Statistical Computing) [42,43]. Blinding the researchers conducting the daily measurements and participants to the study phase was not possible owing to the nature of the intervention.

### Intervention

In our game-based intervention, participants aimed to train selective control of 1 target movement or muscle group, and simultaneously, they tried to reduce the occurrence of involuntary movements or muscle activations around another predefined joint. This training was realized using a technology-based interface in a custom-made game environment. Our flexible setup allowed training a broad selection of target muscles and movements. Together with the patient’s therapist and aligned with the patient’s individual goals, we chose 1 selective target movement, which a participant trained [22].

Movement of the target joint or activity of the target muscle directed an avatar up and down through the game scenario to collect coins and avoid obstacles. With this principle, the player trained the fine-grained muscle activation or movement of the target joint. Involuntary activity in the unwanted muscle or movement in another joint triggered an auditory feedback signal (alarm sound) to inform the player of their occurrence and to inhibit or reduce them. The signal volume reflected the extent of involuntary muscle activity or movements. The game environment also contained elements resembling commercial video games to increase motivation, like progressively unlocking further levels, extra challenges, and character personalization. The game development and study protocol are described in more detail elsewhere [31,32].

We implemented 2 control strategies for the game-based SVMC training. In the first approach, the game was controlled by bending and stretching a joint. Joint movements, that is, joint angles, were captured with inertial measurement units (ArmeoSenso rehabilitation system version 1.0, Hocoma AG). For the second approach, the game was controlled by increasing and lowering the muscle activation without actually moving the joint. sEMG activity was recorded using a varioport device (Becker Meditec). We calibrated the game to the participants’ active range of motion (ROM) or maximal voluntary contraction (MVC), respectively. We originally intended to use the sEMG approach only for more distal target joint movements and the ArmeoSenso for more proximal target joint movements. However, contrary to the pilot trial [31], we encountered issues with the angle recordings of the ArmeoSenso that required frequent recalibrations. Therefore, we favored the sEMG system.

### Primary Outcome Measure

The repeated assessment of SVMC was completed with a short game-based assessment (called a “mini-assessment”) with a comparable setup as the intervention. The participants had to control target joint movements or muscle activity to follow a target line on the screen with an avatar. The target line included predefined up- and downward curves arranged randomly. Like during the training, an auditory feedback signal made the player aware of involuntary movements. We started assessment sessions without preceding training (ie, during the baseline phase and follow-up) with a short accommodation period. The mini-assessment lasted 30 seconds and was repeated 3 times. The actual position of the avatar, the target line, and the feedback signal intensity were recorded in a log file. We provided standardized instructions before starting the assessment. While we considered the instrumented assessment less susceptible to bias, the unblinded assessors did not motivate or give feedback to minimize their influence.

The outcome metrics of the mini-assessment described the target movement accuracy with the root mean squared error (RMSE) between the avatar and the target line and the mean feedback signal intensity to quantify the occurrence of involuntary movements relative to the calibrated ROM or MVC. The combination of these 2 metrics constituted the primary outcome to quantify SVMC. The metrics were calculated with Matlab (Matlab 2020b; The MathWorks Inc) from the mini-assessment log files.

### Further Outcome Measures

Besides our custom SVMC mini-assessment (Figure 1), we also applied a clinical SVMC measure (SCALE for the lower extremities and SCUES for the upper extremities) [21,33]. SVMC of each joint movement was scored on a 3 (SCALE) or 4-point (SCUES) scale. The grading criteria to distinguish between levels of reduced SVMC were based on descriptors for mirror movements of the contralateral joint, movements of another joint apart from the target joint, movement speed, or movements less than the available ROM. The assessments were videotaped and later evaluated by a blinded physical or occupational therapist. Furthermore, we repeatedly assessed the muscle strength of the trained movement with a hand-held dynamometer (microFET 2; Hoggan Scientific). For finger flexion strength, we used a hand grip dynamometer (MAP 80K1S; Kern & Sohn GmbH). We measured in standardized positions and took the mean of 3 repetitions. The WeeFIM, assessed by certified nurses, rated the children’s functional independence in daily life activities [36]. We selected the mobility domain subscore for the lower extremities and the self-care domain subscore (without bladder and bowel items) for the upper extremities. We expressed the scores as a percentage of the maximum domain score.

### Data Analysis and Statistics

#### General

We used R [43] and the additional packages *coin* (version 1.4-2) [44], *mice* (version 3.14.0) [45], *44 nlme* (version 3.1-153) [46], and *relaimpo* (version 2.2-6) [47]. To remove extreme outliers, we excluded strength and RMSE values higher than 3 times the IQR above the upper quartile for each participant individually. For the involuntary movement metric, we used a threshold of 1.5 times the IQR above the upper quartile but did not consider 0 values in the calculation, which otherwise led to very low upper quartiles. We calculated the thresholds once from all data points and once for each phase separately and used the more conservative approach in each case. We averaged the 3 trials of each session and scaled the metrics such that 100% equaled the mean of the last 2 baseline assessments for each individual participant. Scaling facilitated the interpretation of the values and the comparability between participants and allowed to average the accuracy and involuntary movement metrics to yield the primary SVMC outcome. For the involuntary movement metric, we avoided problems with mean values of 0 that would preclude the scaling by first adding the overall mean.

We conducted several analyses. First, the primary analysis investigated the effect of our intervention on the primary outcome with multilevel modeling. Second, we visually analyzed the primary outcome for the individual participants to investigate case-specific effects. Third, we explored which factors were related to the treatment response of the primary outcome and determined their relative importance. Last, we analyzed changes in the outcomes measured at the onset of the baseline, between the phases, at the end of the intervention, and at follow-up.

#### Change in SVMC on the Group Level

In the primary analysis, we investigated the effect of our intervention on the primary outcome with a hierarchical mixed model. It allowed quantitatively summarizing the effects over all cases and accounting for the data structure where repeated measures were nested within cases [48-50]. Our linear mixed model included the fixed and random effects session number (ie, time, coded as 0 for the last session of the baseline phase) and the interaction of the session number with the phase, coded as a binary predictor baseline versus intervention phase. We expected the treatment effect of the intervention to result in a trend change between the phases; thus, our main interest was the interaction coefficient. A negative interaction represents larger improvements during the intervention than during the baseline phase. With the random effects, we acknowledged the individualization of our approach and accounted for the serial dependency of the data. We log-transformed the outcomes to meet the assumptions of the model. We conducted the same analysis separately for the RMSE and involuntary movement metric and the secondary outcome muscle strength (without log-transformation).

#### Change in SVMC on the Individual Participant Level

Second, to visually examine the primary outcome on the patient level, we investigated the individual participant’s treatment response. We descriptively analyzed individual interaction coefficients of the mixed model and complemented them with the result obtained from the robust split middle method [40]. This is a useful visual analysis tool for estimating trend lines within phases, which could be compared to the trend change obtained from the mixed model.

#### Explaining Change in SVMC With Various Characteristics

Third, we explored which factors were related to the treatment response with an analysis of relative importance. We extracted the individual (random) interaction coefficients from the primary model, representing the trend change between phases for each participant. We used these coefficients as the dependent variable of a multiple linear regression model. We included the following predictors: trained side (more affected vs less affected side), involuntary movements (mirror movements vs other involuntary movements), training of an upper versus lower extremity movement, spasticity measured in the trained movement (MAS=0 vs MAS>0), clinical SVMC measure of the trained joint, WeeFIM mobility or self-care percentage score, functional muscle strength (MMT≥3 vs MMT<3), type of brain lesion (congenital vs acquired), topographical distribution (bilateral vs unilateral), and dystonic component as part of the diagnosis versus purely spastic, age, and WeeFIM cognition domain. To quantify the effect of each factor, the amount of explained variance by each factor was determined by a relative importance measure proposed by Lindeman, Merenda, and Gold that considered the order in which the factors were added in the regression by averaging all possible orderings [47]. Missing data among the predictors were imputed with the multiple imputation by chained equations method.

#### Further Outcomes and Follow-Up

Last, changes in the clinical SVMC scores and functional independence were compared between the baseline and intervention phase with the Wilcoxon signed-rank test (handling 0 differences with the Pratt method). The same test was used to compare the follow-up data with the postintervention values (mini-assessment, strength, clinical SVMC score, and functional independence). If a test revealed a significant deterioration, we further tested whether the follow-up values were still better than at the beginning of the study (prebaseline measurements). For the mini-assessment and strength measurements, the postintervention value referred to the mean of the 2 last sessions of the intervention phase, and the prebaseline value referred to the mean of the first 2 baseline sessions. For all paired tests, effect sizes, *r*, were calculated by dividing the *z* value by the square root of the number of observations.

## Results

### Participants

**Figure 2:**
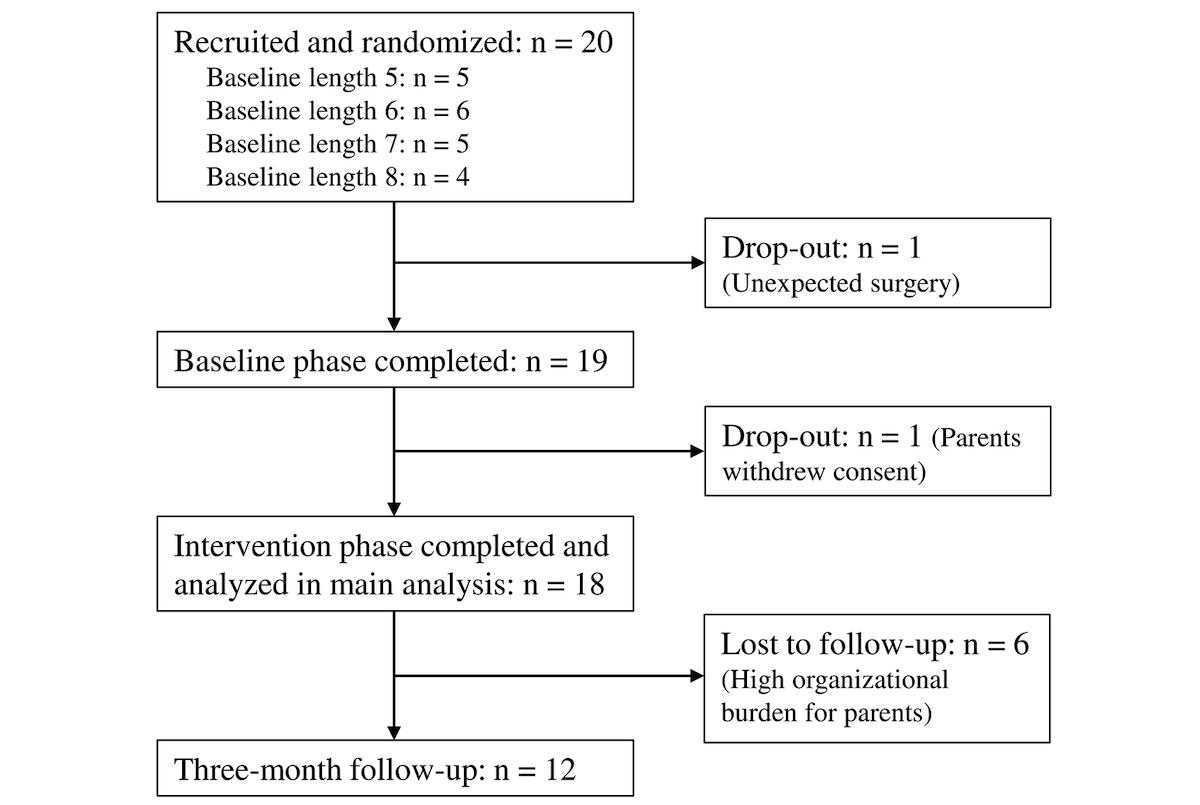
CONSORT (Consolidated Standards of Reporting Trials) flow diagram outlining the number of participants in each phase of the study.

We recruited 20 children and adolescents with impaired SVMC from an upper motor neuron lesion between June 2021 and May 2022. In total, 18 (N=20, 90%) participants completed the SVMC training intervention, while 2 (10%) dropped out before completing at least 8 intervention sessions; 1 participant was excluded from the study because of an unexpected surgery, and the other 1 dropped out from the study because the parents withdrew their consent. A flow diagram of the progress through the phases has been shown in Figure 2.

The 18 participants had a mean age of 12.7 (SD 2.9) years. The majority were diagnosed with spastic CP. The most commonly trained movements were knee extension and finger flexion combined with reducing mirror movements. Further characteristics are shown in Table 1 and Multimedia Appendix 1. All but one participant (ID 5) trained with the sEMG-based system to control the game. Three (n=18, 17%) participants (IDs 1, 5, and 11) missed 1 session in the intervention phase; 2 (11%) because of illness, and 1 (6%) was discharged earlier than planned. The sessions in the baseline phase were, on average, scheduled over a period of 9.6 (SD 3.4) days, and the intervention phase lasted 15.2 (SD 3.4) days.

**Table 1:** Participant characteristics.

ID^a^	Diagnosis	Topography	MACS^b^ or GMFCS^c^	Age (years)	Sex	Trained movement	Trained side	Involuntary movement
1	Spastic CP^d^	Bilateral	GMFCS III	13.8	Female	Knee extension	MA^e^	Mirror movement
2	Stroke (1.5 years)	Unilateral	N/A^f^	10.6	Male	Finger flexion	MA	Mirror movement
3	Spastic CP	Bilateral	GMFCS IV	13.6	Female	Knee extension	LA^g^	Mirror movement
4	Hereditary spastic paraplegia	Bilateral	N/A	16.0	Female	Knee extension	MA	Mirror movement
5	Stroke (1.5 years)	Unilateral	N/A	18.6	Male	Shoulder abduction	MA	Elbow flexion ipsi^h^
6	Stroke (5 weeks)	Unilateral	N/A	10.2	Male	Finger flexion	MA	Mirror movement
7	Spastic CP	Bilateral	GMFCS II	8.4	Female	Ankle dorsal extension	LA	Mirror movement
8	Spastic CP	Unilateral	MACS II	12.4	Male	Finger flexion	MA	Mirror movement
9	Mixed CP (spastic-dystonic)	Bilateral	MACS IV	14.9	Male	Elbow flexion	LA	Shoulder abduction ipsi
10	Spastic CP	Bilateral	GMFCS III	15.8	Male	Knee extension	LA	Mirror movement
11	Mixed CP (spastic-dystonic)	Unilateral	MACS II	12.6	Female	Finger flexion	MA	Mirror movement
12	Spastic CP	Bilateral	MACS III	12.3	Male	Elbow flexion	MA	Shoulder abduction ipsi
13	Spastic CP	Bilateral	GMFCS II	13.5	Male	Knee extension	MA	Mirror movement
14	Spastic CP	Bilateral	MACS III	13.5	Male	Wrist extension	MA	Mirror movement
16	Spastic CP	Bilateral	GMFCS II	13.6	Male	Ankle dorsal extension	LA	Mirror movement
17	Mixed CP (spastic-dystonic)	Bilateral	GMFCS IV	7.0	Male	Knee extension	LA	Mirror movement
19	Meningomyelocele, hydrocephalus	Bilateral	N/A	13.7	Male	Finger flexion	LA	Mirror movement
20	Spastic CP	Bilateral	GMFCS II	9.1	Female	Knee extension	MA	Mirror movement

^a^ID15 and ID18 are not listed because they dropped out.

^b^MACS: Manual Ability Classification System.

^c^GMFCS: Gross Motor Function Classification System.

^d^CP: cerebral palsy.

^e^MA: more affected.

^f^N/A: not available.

^g^LA: less affected.

^h^ipsi: ipsilateral.

### Change in SVMC on the Group Level

**Figure 3:**
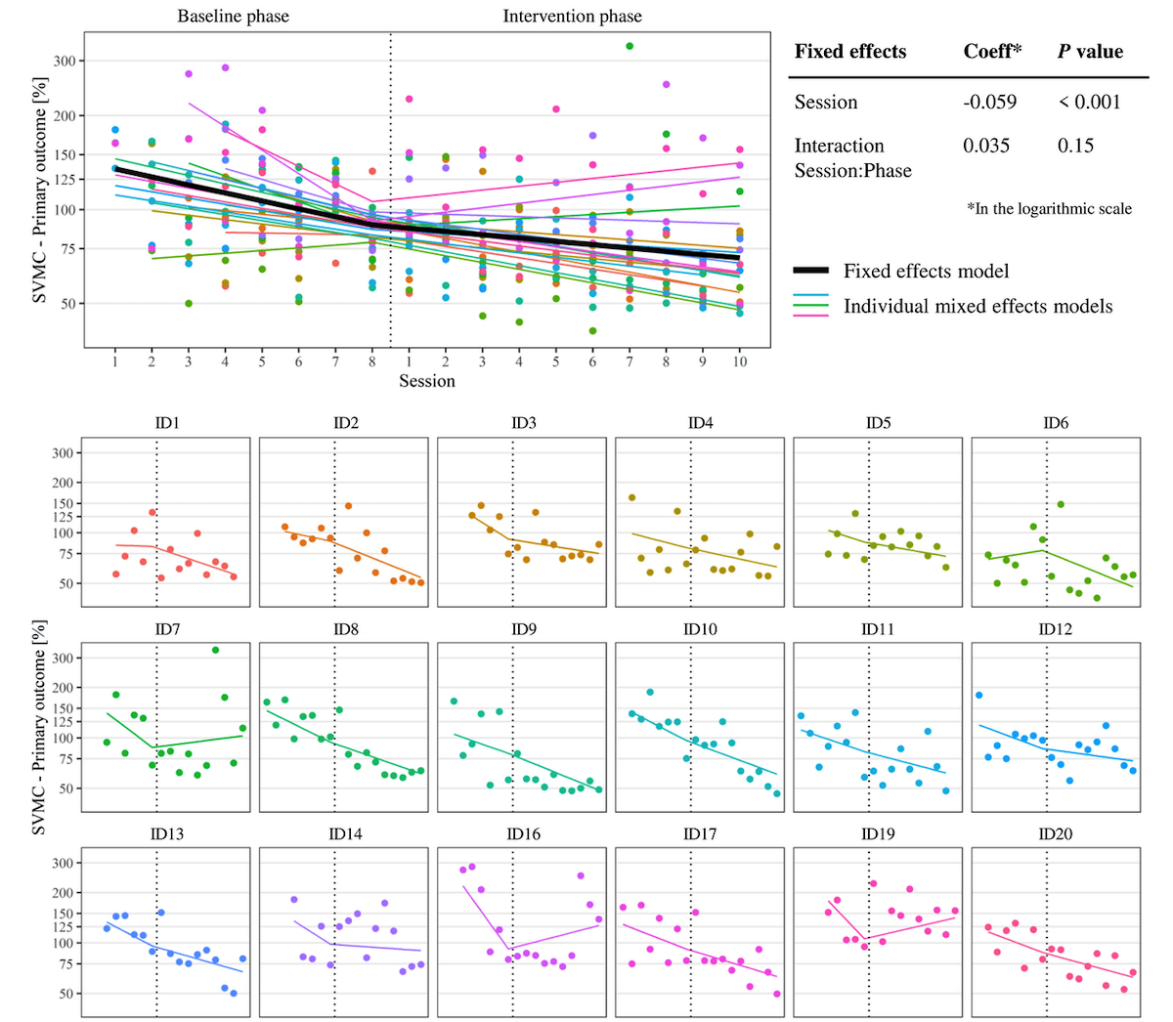
Mixed-effects models for the primary outcome measuring SVMC. Smaller values on the y-axis indicate better performance, the x-axis represents the daily assessment sessions, and each color represents one participant. The panels at the bottom show each participant individually, including the random effects. The top left panel shows the superimposed individual models and the fixed-effects model with the numerical results beside them. Note that IDs 15 and 18 are not included because they dropped out. IDs 1, 5, and 11 missed 1 intervention session. In total, we conducted 875 trials with the mini-assessment, and 20 (2.3%) trials were excluded based on our definition of outliers. coeff: regression coefficient; SVMC: selective voluntary motor control.

The results of the primary linear mixed model are shown in Figure 3. The session parameter indicated a significant (*P*<.001) trend for improvement of the primary SVMC outcome measure during the baseline phase. The interaction coefficient of the session and phase parameter was not significant (*P*=.15), indicating no change in the trend between the baseline and intervention phase (ie, no treatment response). The variation of the random effects between subjects lay between 20% and 30% of the residual variation within participants. A separate mixed model of the accuracy and involuntary movement metric led to the same results, except that the baseline trend was not significant (*P*=.15) for the involuntary movements (Figure S1 in Multimedia Appendix 1).

### Change in SVMC on the Individual Participant Level

In our second case-specific analysis, we could visually identify responders and nonresponders in the individual data (lower panels in Figure 3). Of 18 children, 3 (17%; IDs 1, 2, and 6) showed the desired response (ie, larger improvement during the intervention phase). A roughly continuous trend was present in 7 (n=18, 39%) children (IDs 4, 8, 9, 10,11, 17, and 20). In 8 (n=18, 44%) participants (IDs 3, 5, 7, 12, 13, 14, 16, and 19), the trend change went in the opposite direction (ie, larger improvement during the baseline phase). Visual analysis with the split middle method (Figure S2 in Multimedia Appendix 1) revealed another 3 (n=18, 17%) participants (IDs 5, 10, and 11) who improved more during the intervention phase. The split middle analysis confirmed the response patterns of the remaining children with similar trends between the phases or a trend change opposite to what was expected.

### Explaining Change in SVMC With Various Characteristics

**Figure 4:**
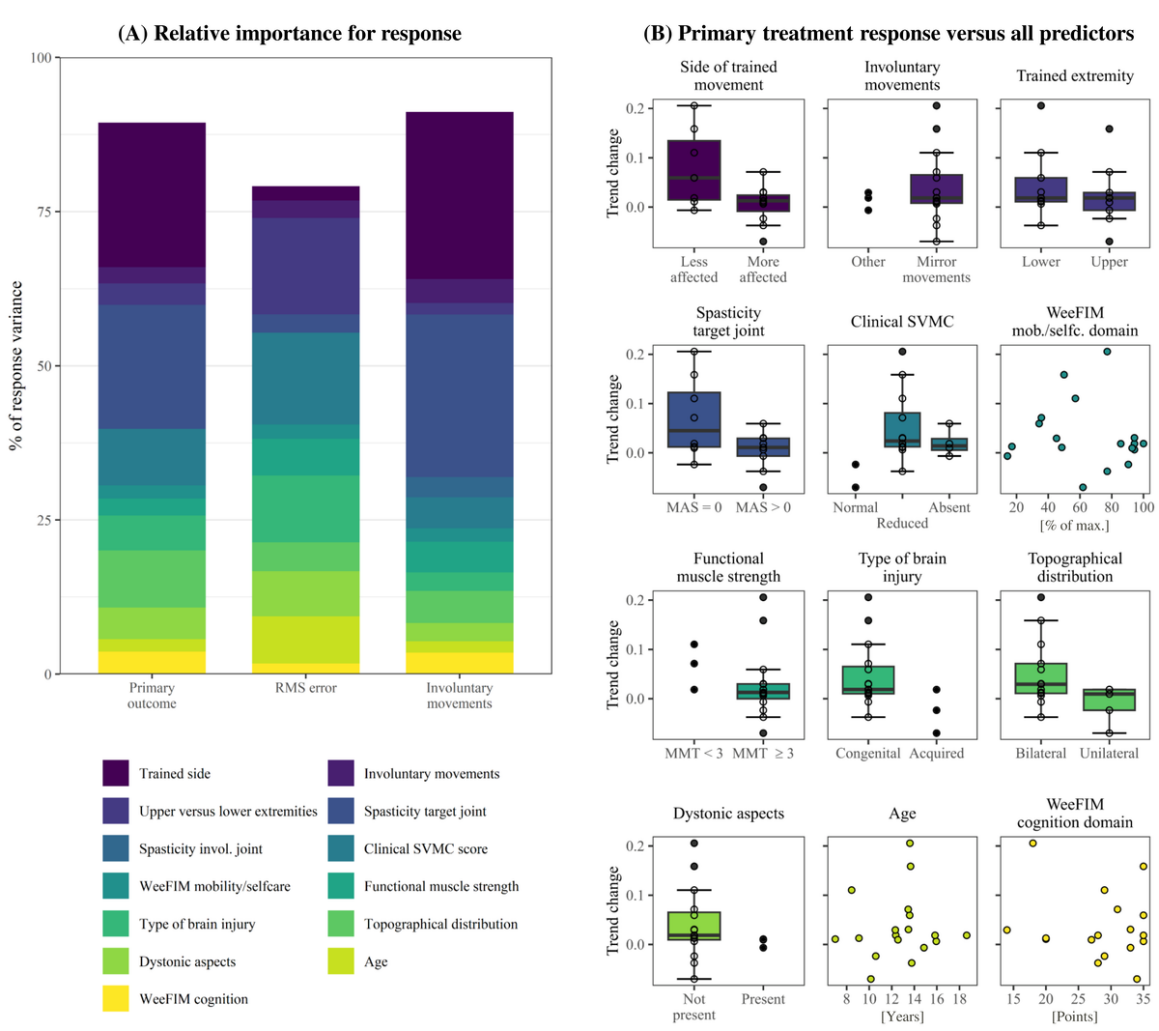
Exploration of treatment response predictors. (A) Each color represents the relative importance of a predictor for explaining the individual treatment response to the game-based SVMC training in a multiple linear regression model for the primary outcome (left bar) and 2 metrics of the mini-assessment separately (middle bar: target movement accuracy and right bar: involuntary movements). (B) Visualization of the direction of the predictors’ effects on the treatment response in the primary outcome for each predictor. The individual treatment response is measured by the trend change between the baseline and intervention phases, which is represented by the individual interaction coefficient. A negative trend change indicates a larger improvement during the intervention phase. MAS: Modified Ashworth Scale; MMT: Manual Muscle Test; mob.: mobility; selfc.: self-care; SVMC: selective voluntary motor control; WeeFIM: functional independence measure for children.

Our third analysis, the multiple linear regression models predicting the individual treatment effect, revealed that we could explain 75% and more of the response variance (Figure 4). The most important predictors of more favorable treatment response of the primary outcome were training the more affected side (23.5%), spasticity in the trained movement (20.1%), a unilateral brain lesion (9.2%), and a better score in the clinical SVMC measure (9.1%). The 2 most relevant factors were the same for the involuntary movement metric (27.1% and 26.4%, respectively). For the RMSE, the highest percentages of explained variance were attributed to the factors training a lower extremity movement (15.7%), better clinical SVMC measure (14.9%), and acquired brain injury (10.8%).

### Further Outcomes and Follow-Up

For muscle strength, none of the parameters from this linear mixed model were significant. There was a slight trend for strength improvements during the baseline phase (session coefficient=0.63, *P*=.57), amplified during the intervention phase (interaction=1.25, *P*=.25). For most participants, the clinical SVMC score for the trained movement did not change throughout the study. If there were changes, we observed improvements during the baseline phase or deterioration during the intervention phase, resulting in significantly different changes between the phases (*P*=.008) in the opposite direction than expected (Table 2). The changes in functional independence did not differ between the phases.

The primary outcome had significantly deteriorated at follow-up compared to postintervention values (*P*=.03; Table 2). Therefore, we additionally compared the follow-up to preintervention scores. The median follow-up score was still better than at the beginning of the baseline, and the corresponding statistical test nearly reached significance (*P*=.05, *r*=−0.40). The deterioration at follow-up compared to postintervention values was mainly driven by the involuntary movement metric (Table 2). Further comparison showed that despite a considerable difference between the median follow-up (71.2) and baseline scores (110.5), these were not statistically different (*P*=.73, *r*=−0.07) because of 2 extreme cases. Muscle strength and functional independence had further increased during the follow-up period, while the clinical SVMC score did not change (Table 2).

**Table 2:** Descriptive statistics (median and IQR) of the outcomes at the beginning and end of each study phase and at 3-month follow-up and comparison of the changes between phases.

	Prebaseline value^a^, median (IQR)	Preintervention value^a^, median (IQR)	Postintervention value^a^, median (IQR)	Baseline vs intervention change			Follow-up value, median (IQR)		Postintervention value vs follow-up	
				*P* value	*r*			*P* value		*r*
Primary outcome	125.3 (108.9-136.0)	100 (—^b^)^c^	64.2 (58.1-73.2)	LMM^d^	LMM	71.7 (60.1-97.7)		.03		0.43
Root mean squared error	116.7 (96.3-163.5)	100 (—)	68.4 (61.7-80.4)	LMM	LMM	73.5 (68.1-89.2)		.62		0.10
Involuntary movements^a^	110.5 (78.8-147.0)	100 (—)	56.9 (44.6-69.9)	LMM	LMM	71.2 (51.1-96.8)		.03		0.45
Dynamometry^a^	95.0 (86.6-102.0)	100 (—)	115.7 (106.2-125.0)	LMM	LMM	127.9 (112.6-140.1)		.02		0.44
Clinical selective voluntary motor control test (in points)	1 (1-2)	2 (1-2)	1 (1-2)	.008	0.44	2 (1-2)		>.99		0.00
WeeFIM (functional independence measure for children)^e^	69.5 (46.1-92.3)	75.7 (50.7-92.9)	81.7 (53.6-93.9)	.14	−0.25	93.6 (79.8-100.0)		.008		0.54

^a^For the repeatedly assessed outcomes, pre- and postmeasurement values of each phase equal the mean of the first or last 2 points of the phase.

^b^Not applicable.

^c^Scaled such that 100% equaled the mean of the last 2 baseline assessments.

^d^LMM: analyzed with the linear mixed model; the results are presented separately in the manuscript.

^e^Expressed as a percentage of the maximum domain score.

## Discussion

### Principal Findings

This study evaluated the effectiveness of a novel game-based intervention to specifically improve SVMC in children with upper motor neuron lesions. The game-based SVMC training aimed to improve the accurate control of a target movement while simultaneously reducing involuntary movements with the help of auditory feedback. With a randomized multiple baseline design, we compared changes in SVMC between the baseline phase and intervention phase, when complementing regular inpatient rehabilitation with 10 SVMC training sessions. A linear mixed model revealed a significant trend for improvement of the primary outcome already in the baseline phase. This trend continued in the intervention phase but did not differ from the baseline, suggesting no response to the SVMC training. A multiple regression analysis revealed that participant and training characteristics could explain a large proportion of the response variation between the children.

### Change in SVMC and Prior Studies Targeting Improvements in SVMC

Training approaches that aim to improve SVMC are rare and often target only a specific aspect of SVMC [13]. For example, a robot-assisted ankle training program included playing computer games by graded assisted or resisted ankle movements. The intervention focused on improving motor control of the target joint in children with uni- or bilateral CP (GMFCS I-III) in combination with passive stretching [14]. Involuntary movements were not addressed with this intervention. Nevertheless, after eighteen 1-hour training sessions in 6 weeks, the trained ankle and leg SCALE scores improved significantly. Two other studies that used the same intervention and similar protocol confirmed SCALE improvements of the trained leg in different settings (eg, home-based training) [51,52]. Besides improvements in the SCALE, all 3 studies [14,51,52] measured a simultaneous increase in the active ROM or dorsal extension strength, maybe because the ankle training robot in some training phases also exerted resistance against the participants’ movements. Thus, improvements in the SCALE were likely due to changes in the descriptor ROM, which seems more related to strength than SVMC, and not descriptors regarding movements of other joints. It is also interesting to note that children with more impaired SVMC were more likely to improve, in contrast to our results. Seven of 9 children who improved their ankle SCALE score had 0 points at baseline. Moreover, of the 5 children with 1 SCALE point at baseline, only 2 could improve [14]. Favoring more impaired children might result from the intervention that included sequences where the robot actively assisted movements.

Training selective muscle activation was the focus of “NeuroGame” therapy. A computer game was controlled by sEMG signals of 2 muscles, including activating a target and relaxing another muscle. The first study aimed to reduce cocontraction of the wrist flexors and extensors in children with unilateral spastic CP (MACS II-III) [17]. Throughout the 5 weeks of training for 5 to 13 hours, 3 of 4 children could increase the phases of selective agonist activity during the game, that is, without simultaneous antagonist activity. However, these improvements were not reflected by the clinical outcomes, which showed variable and unclear results. In the second study, children with bilateral spastic CP (GMFCS I-III) practiced the isolated activation of the tibialis anterior muscles with the computer game [18]. They had trained at home for 1 to 9 hours over 6 weeks. The overall SCALE score improved by at least 1 point in 6 of 9 children. However, whether the improvement arose from the trained ankle joint or other joints is unclear.

Araneda et al [53] concluded that there was a significant reduction of mirror movements after intensive bimanual training (90 hours over 2 weeks) in children with unilateral CP (MACS I-III). Similarly, Adler et al [54] investigated the reduction of mirror movements of the hands in children with unilateral CP (MACS I-III). The intensive 3-week therapy program (4 hours/day for 13 days) consisted of bimanual activities and simple exercises explicitly focusing on reducing mirror movements. While the children improved their bimanual performance, the occurrence of mirror movements remained unchanged. Therefore, the authors hypothesized that the neurological origin of mirror movements could not be changed. Still, children could learn to control the influence of mirror movements when they focused on them. In light of these findings, the challenge of our approach was to focus on 2 goals simultaneously, that is, accurately moving the target joint and reducing involuntary movements. Unfortunately, this caused the participants to sometimes prioritize 1 task over the other, increasing the variability of the outcome metrics.

These studies [14,17,18,51,52,54] have in common that the interventions were not compared against a control group or (equally intensive) condition. In our study, we implemented the baseline phase as a control and analyzed the differences between the phases. We did not detect a difference between the baseline and intervention phases. If we had included and analyzed only the intervention phase, as these studies did, we would also have reported improvements in the primary outcome. We can only speculate why we did not observe a trend change between the phases. First, the baseline trend was unexpected because the feasibility study [31] showed no improvements over several repetitions of the mini-assessment within 1 session. Furthermore, a pilot measurement of a 5-day baseline phase in a patient aged 13 years with unilateral CP indicated stable outcomes. While we cannot exclude that the participants improved during the baseline phase because of further familiarization with the mini-assessment, we assume that the effects might be caused or enhanced by the concomitant therapies. Although the regular therapy sessions did not target SVMC specifically, exercises for improving coordination or motor control could increase the accuracy component of the SVMC assessment. Indeed, therapists reported that for 6 (33%) of 18 children, their therapy toward the rehabilitation goal included motor control training. However, during regular treatment, less training was provided for reducing involuntary movements. Second, the decreased motivation and energy of the participants could have flattened the trend in the intervention phase because, for many patients, the end of the study coincided with the end of the rehabilitation stay. However, we do not expect that fatigue flattened the trend in the intervention phase because participants conducted the mini-assessment after practicing. Indeed, we repeated the mini-assessment on another day (during a further visit for other postassessment measurements) after the last training session from participant 9 (ID 9) onward, and the outcomes were not different.

Further and more detailed analyses of each component of the primary outcome indicated that improvements were mainly based on improving motor control while the participants could not learn to inhibit involuntary movements. We noted a clear improvement in accuracy for all participants in both phases. This could explain why we also observed improvements in the SVMC outcome measure during the baseline phase because regular rehabilitation also included motor control training. At the same time, the involuntary movement metric showed much more variable response patterns both within- and between participants (Figure S1 in Multimedia Appendix 1). Controlling or reducing the occurrence of involuntary movements appeared difficult, as already noted by Adler et al [54].

### Explaining Change in SVMC With Various Characteristics

We evaluated which factors were predictive of a favorable individual treatment response. One factor was a better score in the clinical SVMC measure at baseline, indicating that children with minor impairments in SVMC can benefit more from our game-based intervention than children with more pronounced SVMC impairments. Contrary to Adler et al [54], Araneda et al [53] found a reduction in mirror movements after bimanual training. Apart from differences in the therapy content and a higher total dose in the second study, these children also presented less pronounced mirror movements. This disagreement could be further related to a different reorganization or organization of the corticospinal tract. Children with bilateral projections might benefit from therapy-induced changes to interhemispheric inhibition as opposed to children with ipsilateral projections. One study investigating brain activation during ankle dorsi- and plantar flexion in 9 children with bilateral spastic CP (GMFCS II-V) revealed that SCALE scores correlated positively with activity in the primary motor and sensory cortices and negatively with cerebellar activity [55]. Based on this study, we could speculate that depending on how much SVMC was impaired, other brain areas with differing adaptive capabilities were involved.

A second predictor was that children training their more affected side responded better. One apparent reason could be that this side had a higher potential for improvement. This does not contradict the previous predictor, that is, better SVMC, because more versus less affected is only a relative comparison within a child and should be distinguished from better or worse absolute SVMC scores between children. We usually decided to train the less affected side in children with pronounced impairments in SVMC. In other words, these children trained their “less affected” joints, which were likely still more affected than the “more affected” joints of children with generally minor impairments in SVMC.

As a third predictor, children showing an increased muscle tone (MAS>0) in the trained movement were better responders. Similarly, the response to NeuroGame for isolated tibialis activation seems lesser for children with lower ankle spasticity scores [18]. Furthermore, all participants in the robotic ankle training studies presented ankle spasticity [14,51]. An increased muscle tone can be regarded as an additional challenge for a child but also as another opportunity for improvement. We observed children having difficulties relaxing the target muscle once activated, causing initially large accuracy errors in the mini-assessment. However, learning to stay more relaxed already improved the outcome before actually training a graded activation to improve further accuracy. It would have been interesting to know whether there were indeed changes in muscle tone, as reported by others [14,52], but we did not repeat the MAS at the end of the intervention. Finally, these factors explaining the highest percentage of the individual treatment response could serve to refine the inclusion criteria for future trials and identify responders to treatment.

### Further Outcomes and Follow-Up

The clinical SVMC assessment scores of the target movement were stable for most participants, as expected. However, if there were changes, they were in the opposite direction than expected. We might explain improvements in SCALE or SCUES scores during the baseline phase by an increased focus of attention on involuntary movements that had emerged over the baseline sessions. Receiving immediate feedback on involuntary movements (during the mini-assessment) could be a stronger incentive to inhibit involuntary movements than the instruction during the clinical assessment during the first visit not to move other joints. Nevertheless, the observed changes of 1 SCUES point for single joints exceeded the smallest detectable change, which has been set at a value slightly below 1 point, which is an impossible score [33]. For the SCALE, these thresholds have yet to be investigated.

Despite the worsening of the mini-assessment metrics at the follow-up compared to postintervention measurements, the median values, the statistical testing (*P*=.052), and the moderate effect size indicated a trend that SVMC was still better than at the onset of the trial. On the one hand, there is a partial maintenance of improved selectivity at 3-month follow-up. In contrast, this may indicate that the positive effects on SVMC could diminish without appropriate ongoing intensive therapy. The issue with this analysis was that the power to detect changes in the follow-up analysis was limited because 6 (33%) of 18 participants were lost to follow-up. This was mainly caused by difficulties in scheduling the appointment because of the high organizational burden for the parents. Furthermore, as the WeeFIM assesses activities in daily life, we need to consider that children were evaluated at the rehab clinic during the study and at home during follow-up. As the daily life performance of children depends on their environment, the different settings might have influenced the scoring.

### Limitations

As the trial did not show the expected results, we should critically reflect on our study. We designed it to best address the challenges of conducting a clinical trial within our setting, where the participants attend a multimodal rehabilitation program concomitant to the SVMC training. We had a heterogeneous study population representing the children treated daily in rehabilitation clinics and selected an individualized approach tailored to the needs of the participants (eg, selection of the trained movement). Protocols that resemble clinical practice may facilitate the translation of the findings [41].

The measurement of stable outcomes in the baseline phase would be desirable, but we observed a trend and considerable variability. Although we had an accommodation period before we started with the mini-assessments, this period should be extended, and each phase should include more data points. While this would statistically decrease the variability, it would prolong the study beyond most children’s rehabilitation stays. In addition, we thought the SVMC training was so specific and intensive that it would exceed the effects of the regular rehabilitation program. Consequently, we underestimated the impact of other therapies on motor control and coordination, which likely caused the trend during the baseline phase. Although the effects of ongoing therapies could also have confounded the follow-up results, particularly if participants had continued with the intensive inpatient rehabilitation program, we could not identify any trends. Of the 12 participants with follow-up assessments, 2 (17%) were still inpatients at the time of follow-up; one of them improved and the other worsened in SVMC compared to the posttreatment assessment. Three (n=12, 25%) participants remained inpatients for 1 to 3 weeks before being discharged; 1 improved, 1 deteriorated markedly, and 1 slightly worsened comparable to the group average pattern. Seven (n=12, 58%) participants were discharged shortly after completion of the SVMC interventions; 1 improved, 1 worsened considerably, and 5 showed a small deterioration.

A problem with the mini-assessment was the unbalanced visualization of the 2 components of SVMC. Participants saw the movement accuracy directly on the screen, and the accuracy was reflected in the “game score.” However, the involuntary movements were represented by the auditory feedback signal, which might have been less accessible and was sometimes even ignored. Thus, the incentive to reduce involuntary movements might have been lower than moving accurately. Furthermore, we had not evaluated the mini-assessment extensively for validity and reliability in the form used in this study; it was an advancement of a game-based SVMC assessment [25] for which we have investigated the psychometric properties extensively [56,57]. Similar to our current observations, this study suggested that the occurrence of involuntary movements might be variable because the absolute reliability of the involuntary movement measure was lower than the accuracy of the target movement.

After the blinded rating of the SCUES at the end of the study, it turned out that 2 (11%) of the 18 participants had scored 3 points at baseline, that is, normal SVMC for the target movement, which would violate the inclusion criterion. The mirror movements were not visible in the clinical SVMC test. However, they were well observed by their occupational therapist during therapy and documented by the mini-assessment. Therefore, the participants remained in the study. We recognized that the SCUES assessment was not sensitive enough to serve as an inclusion criterion for reduced SVMC in these 2 cases.

The methodological considerations and the observation that improvements were present in both phases and that these were mainly driven by the accuracy component of SVMC could lead to future work focusing only on the motor control aspect of SVMC. In addition, a more task-oriented and functional approach could be considered, similar to the regular rehabilitation program in this study, which is likely to have caused the improvements in motor control (during the baseline phase). This approach aligns with therapy trends for children with CP, which have moved toward practical interventions and functional performance rather than restoring physiological movement patterns (such as targeting SVMC) [58].

### Conclusions

On a group level, the primary SVMC outcome measure improved during the baseline and intervention phases. Thus, the regular rehabilitation program already led to improvements in SVMC, although it did not target to enhance SVMC, and the game-based SVMC training could not show additional improvements. At the same time, the variability within and between participants was huge, making inferences difficult. Interestingly, a large part of the response variability could be explained by several characteristics that can determine whether a patient was likely to benefit from the intervention. This latter finding could be valuable to identify potential responders for therapy and design future trials targeting improvements in SVMC.

## Appendix

Multimedia Appendix 1 Supplementary figures and tables including the mixed-effects models for the 2 components of the primary outcome, the split middle and the response predictor analyses.

Multimedia Appendix 2 CONSORT (Consolidated Standards of Reporting Trials) checklist.

## Abbreviations

CP: cerebral palsy
GMFCS: Gross Motor Function Classification System
MACS: Manual Ability Classification System
MAS: Modified Ashworth Scale
MMT: Manual Muscle Test
RMSE: root mean squared error
ROM: range of motion
SCALE: Selective Control Assessment of the Lower Extremity
SCUES: Selective Control of the Upper Extremity Scale
sEMG: surface electromyography
SVMC: selective voluntary motor control
WeeFIM: functional independence measure for children

## Data Availability

The datasets generated and analyzed during this study are not publicly available owing to ethical concerns about the small number of patients but are available from the corresponding author upon reasonable request.
