# Supplementary material for: Effectiveness of Game-Based Training of Selective Voluntary Motor Control in Children With Upper Motor Neuron Lesions: Randomized Multiple Baseline Design Study
Source: JMIR Form Res. 2024 Nov 18;8:e47754. doi: 10.2196/47754 (PMC11612595; doi:10.2196/47754)
Supplement: Multimedia Appendix 1 [file formative_v8i1e47754_app1.pdf]

Accuracy – RMS error

Fixed effects	Coeff*	*P* value
Session	-0.049	< 0.001
Interaction Session:Phase	0.017	0.19

Involuntary movements

Fixed effects	Coeff*	*P* value
Session	-0.050	0.15
Interaction Session:Phase	0.028	0.56

*In the logarithmic scale

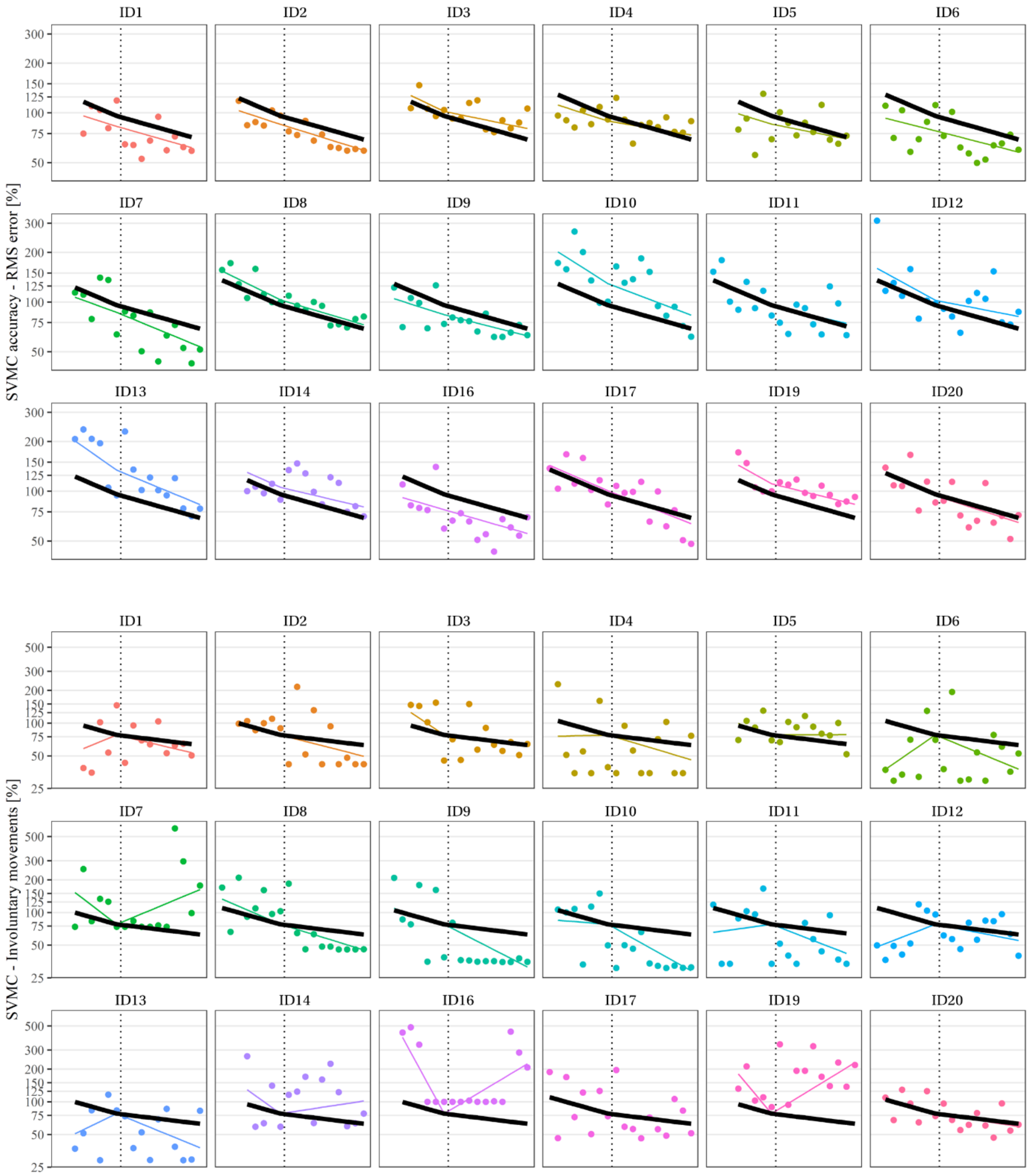

Figure S1: Mixed effects models for the two components of the primary outcome. Smaller values indicate better performance and each color represents one participant. The tables at the top indicate the numerical results. The panels show all participants separately (fixed effects and individual random effects model). The upper rows correspond to the RMS error while the bottom rows indicate the involuntary movement metric.

Abbreviations: coeff: regression coefficient, RMS: root mean squared, SVMC: selective voluntary motor control.

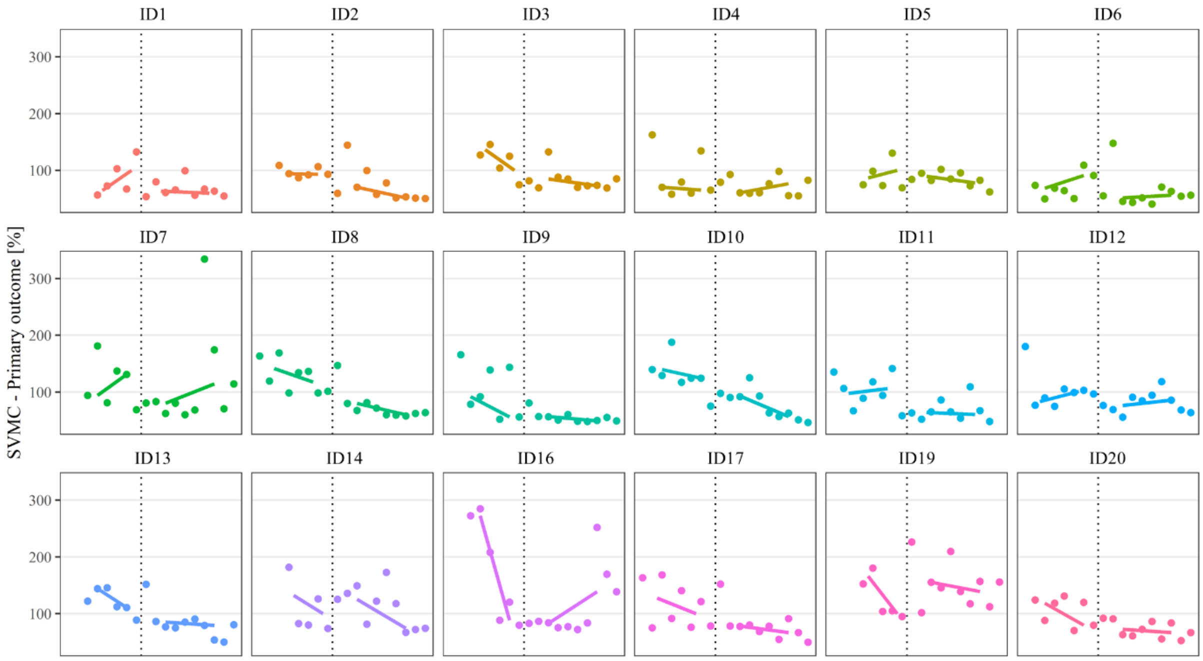

Figure S2: Individual data with trend lines for each phase determined by the split middle method. Smaller values indicate better performance and each color represents one participant.

Abbreviations: SVMC: selective voluntary motor control.

Table S1: Participant characteristics – related to the predictor analysis

ID	More affected side	MAS [x/4]	MMT [x/5]	SCALE [x/2] or SCUES [x/3]	WeeFIM mobility or self-care [% of max]	WeeFIM cognition [x/35]
1	Left	1	5	1	77.1	28
2	Left	0	5	3	90.5	29
3	Right	1	4	0	34.3	35
4	Left	1	5	1	94.3	35
5	Right	n/a	2	1	85.7	28
6	Right	1	4	3	61.9	34
7	Right	0	2	1	57.1	29
8	Right	0	4	2	100.0	n/a
9	Right	1+	5	1	14.3	33
10	Left	1	5	1	94.3	35
11	Left	0	4	1	92.9	27
12	Left	1	4	2	45.2	14
13	Right	1	3	1	94.3	33
14	Left	0	2	2	35.7	31
16	Right	0	5	1	77.1	18
17	Right	1	3	1	48.6	20
19	Left	0	5	2	50.0	35
20	Left	0	4	1	17.1	20

Among the data included the predictor analysis, two values out of 288 (< 1%, 13 predictors + 3 outcomes x 18 participants) were missing and imputed with the multiple imputation by chained equations method. In one case, the MAS was not tested for medical reasons and in the other case, we could not assess the WeeFIM cognition due to organizational issues.

Abbreviations: MAS: modified Ashworth scale, MMT: manual muscle test, n/a: not available, SCALE: selective control assessment of the lower extremity, SCUES: selective control of the upper extremity scale, WeeFIM: functional independence measure for children.
